# The Toxic Effects of Pathogenic Ataxin-3 Variants in a Yeast Cellular Model

**DOI:** 10.1371/journal.pone.0129727

**Published:** 2015-06-08

**Authors:** Marcella Bonanomi, Cristina Visentin, Gaetano Invernizzi, Paolo Tortora, Maria Elena Regonesi

**Affiliations:** 1 Department of Biotechnologies and Biosciences, University of Milano-Bicocca, Milan, Italy; 2 Milan Center of Neuroscience (NeuroMI), Milan, Italy; 3 Department of Statistics and Quantitative Methods, University of Milano-Bicocca, Milan, Italy; Universitat Autònoma de Barcelona, SPAIN

## Abstract

Ataxin-3 (AT3) is a deubiquitinating enzyme that triggers an inherited neurodegenerative disorder, spinocerebellar ataxia type 3, when its polyglutamine (polyQ) stretch close to the C-terminus exceeds a critical length. AT3 variants carrying the expanded polyQ are prone to associate with each other into amyloid toxic aggregates, which are responsible for neuronal death with ensuing neurodegeneration. We employed *Saccharomyces cerevisiae* as a eukaryotic cellular model to better clarify the mechanism by which AT3 triggers the disease. We expressed three variants: one normal (Q26), one expanded (Q85) and one truncated for a region lying from the beginning of its polyQ stretch to the end of the protein (291Δ). We found that the expression of the expanded form caused reduction in viability, accumulation of reactive oxygen species, imbalance of the antioxidant defense system and loss in cell membrane integrity, leading to necrotic death. The truncated variant also exerted a qualitatively similar, albeit milder, effect on cell growth and cytotoxicity, which points to the involvement of also non-polyQ regions in cytotoxicity. Guanidine hydrochloride, a well-known inhibitor of the chaperone Hsp104, almost completely restored wild-type survival rate of both 291Δ- and Q85-expressing strains. This suggests that AT3 aggregation and toxicity is mediated by prion forms of yeast proteins, as this chaperone plays a key role in their propagation.

## Introduction

The expansion of an unstable translated CAG repeat causes at least ten dominantly inherited neurodegenerative disorders known as polyglutamine (polyQ) diseases. These include Huntington disease, spinal and bulbar muscular atrophy, dentatorubropallidoluysian atrophy, and seven autosomal dominant spinocerebellar ataxias (SCA1, 2, 3, 6, 7, 12 and 17) [1–3]. In all these diseases, a polyQ stretch expanded beyond a critical threshold leads to misfolding of the respective protein, its aggregation into large intracellular inclusions, cytotoxicity and finally dysfunction and demise of specific neurons [4]. The loss of function resulting from misfolding might also be involved in the mechanisms of pathogenesis [5,6]. Machado-Joseph disease, otherwise known as spinocerebellar ataxia type-3 (SCA3), is the most common form of autosomal dominantly-inherited ataxia, and characterized by pyramidal symptoms associated in varying degrees with a dystonic-rigid extrapyramidal syndrome or peripheral amyotrophy [7,8]. The gene causatively associated with SCA3 is *atxn3*, which encodes a polyQ-containing protein known as ataxin-3 (AT3) [9]. This is a conserved and ubiquitous protein with a wide distribution in the brain, although different regions present varying expression levels [10]. AT3 is composed of a structured globular N-terminal domain, the Josephin domain (JD), which displays ubiquitin hydrolase activity, followed by a disordered C-terminal tail containing two ubiquitin-interacting motifs (UIMs) and the polyQ stretch of variable length, whose expansion beyond a certain threshold triggers SCA3 [11,12]. To date, the mechanism by which polyQ-expanded AT3 leads to SCA3 pathogenesis has not been fully clarified. It has been largely reported that the polyQ expansion induces transition to aggregation-prone conformations [13–15]. As for most amyloid-forming proteins, several pathways may drive the conversion of the soluble protein to amyloid aggregates, although small aggregates and oligomers are the species responsible for cytotoxicity [16–19]. It has been suggested that the soluble amyloid oligomers have common mechanisms of toxicity [20], for example being able to destabilize the cellular membrane or to sequester quality control system components and transcription factors, causing proteotoxic stress and transcriptional dysregulation [21–23]. Consequently, this investigation is aimed at clarifying the mechanisms underlying AT3 aggregation and how the different protein variants exert their cytotoxicity. To provide insight into this issue, we have used the budding yeast *Saccharomyces cerevisiae*, an eukaryotic model organism widely used in studies on neurodegenerative diseases [24] including the case of polyQ toxicity and aggregation [25] despite the lack of any AT3 homologous in yeast. Nevertheless, most processes involved in neurodegenerative disorders such as apoptosis and necrosis [26], mitochondrial damage, oxidative stress, protein aggregation and degradation can be analyzed within yeast [27]. Models of protein aggregation disorders in *S*. *cerevisiae* have provided new insight into Parkinson’s disease [28,29], amyotrophic lateral sclerosis [30,31], and Huntington’s disease [32–34]. Also, nucleocytoplasmic shuttling activity of AT3 has been investigated, which showed active import and export from the nucleus [35]. Here, we have characterized the mechanisms of toxicity exerted by AT3 variants: one normal (AT3-Q26), one expanded-pathological (AT3-Q85), and one truncated for a region lying from the beginning of its polyQ stretch to the end of the protein (AT3-291Δ). Normal AT3 variants have polyQs in the range 10–51; expanded, pathogenic ones, 55–87 [36]. All the proteins were expressed in fusion with the green fluorescent protein (GFP) at the C-terminus. First, we have shown that the expression of the expanded form causes a significant viability reduction compared with the normal, wild type strain. We have demonstrated that the toxicity is associated with an accumulation of reactive oxygen species (ROS), an increase of catalase (CAT) activity, an alteration in the balance of reduced glutathione (GSH) and an induction of necrosis. We have assayed the truncated variant to assess the role of the protein context in polyQ toxicity. Actually, we have previously demonstrated the toxic effects of this truncated form in *Escherichia coli* [37]. Here, we demonstrate an effect also on yeast cell growth and some markers of toxicity in a way comparable to that of the full-length, expanded form. This implies that AT3 regions outside the polyQ tract could also determine its pathological features.

## Materials and Methods

### Yeast strains and plasmids

Experiments were carried out in W303 (*MATα can1-100 ade2-1 his3-11*, *15 trp1-1 ura3-1 leu23*,*112*) yeast strain. p426GALhtt103QGFP plasmid (Addgene) [38] was digested with *BamHI* restriction enzyme to excise huntingtin gene. AT3-Q26, AT3-Q85 and AT3-291Δ genes were digested with *BamHI* and the resulting fragments were subcloned into the digested plasmid in frame with GFP protein at the C-terminus. Transformation of yeast was performed by the lithium acetate method [39]. Yeast cells transformed with the p426GAL empty vector were used as a control.

### Yeast growth conditions

Cells were grown overnight in selective media containing glucose (2%), washed three times in sterile water and diluted to an OD_600_ of 0.1 in selective media containing 2% galactose as inducer of AT3 expression. Analyses were performed after 16, 24 or 48 h of induction, unless otherwise noted.

### Confocal microscopy analysis of protein aggregation

Fluorescence microscopy was performed to detect protein aggregation using a Leica Mod. TCS-SP2 confocal microscope (Leica Microsystem, Wetzlar, Germany) and the fluorescence of GFP was excited with the 488 nm line.

### Clonogenic growth assay

About 100 cells derived from the overnight cultures were grown in the presence or the absence of one of the following: i) 100 μM tetracycline, ii) 100 μM epigallocatechin-3-gallate (EGCG), iii) 5 mM guanidine hydrochloride (GuHCl). Then, cultures were washed in water, spread on a plate with selective medium containing glucose as the sole carbon source and on another plate with selective medium containing galactose as the sole carbon source. The colony-forming ability was plotted as the ratio of the number of cells grown on galactose to those grown on glucose and expressed as percentage.

### Filter trap assay and dot blot analysis

For each strain, 1 ml of culture was harvested after 24 h of induction. Total protein extracts were obtained as previously described [40] and the concentration in different samples were determined by the Bradford assay (Coomassie Plus Protein Assay Reagent, Thermo Scientific, Rockford, IL, USA). Equal amounts of the different samples were subjected to either a filter trap assay or dot blot analysis as previously described [41], using an anti-AT3 Z46 rabbit polyclonal primary antibody [42] and anti-rabbit fluorescent secondary antibody (Donkey anti-rabbit IRDye 800 CW, Li-Cor, Lincoln, USA). Dot blots were also performed, using OC antibodies that specifically recognize soluble fibrillar oligomers [43]. Western blotting was performed using primary anti-AT3 Z46 and anti-rabbit fluorescent secondary antibody. Membranes were imaged using a LiCor Odyssey Fc scanner.

### MTT assay

The MTT [3-(4,5-dimethylthiazoyl-2-yl) 2,5-diphenyltetrazolium bromide] assay was performed as described by Teparić [44] with minor modifications. The assay quantifies the capability of actively respiring cells to reduce the water-soluble MTT to an insoluble purple formazan. Cells from 1 ml of culture were harvested and resuspended in 0.4 ml 5 μg/mL MTT. The mixture was incubated at room temperature under shaking for 2 h. Then, cells were harvested and resuspended in 1 ml acid 2-propanol (0.04 M HCl in 2-propanol). The suspension was shaken for 10 min and then centrifuged at 7000 x *g* for 10 min. OD_540_ of the supernatant was measured. Data were expressed as percentage of MTT reduction with respect to the control.

### ROS assay

H_2_O_2_ levels were monitored using the Red Hydrogen Peroxide Assay Kit (Enzo Life Sciences) according to the manufacturer’s protocol. Cells from 3 ml of culture were harvested and resuspended in lysis buffer (20 mM phosphate buffer, 5 mM EDTA, 0.2 mM PMSF, pH 7.2). Cells were broken using glass beads (0.5 mm diameter) by vortexing five times for 1 min with intervals of 1 min on ice. Cell debris was pelleted and the supernatants used for the test. The conversion of red peroxidase substrate to resorufin was determined measuring OD_576_. Data were expressed as fold increase with respect to the empty vector strain level.

### Determination of glutathione levels

Reduced (GSH) and total glutathione content was determined by the method of Boyne and Ellman [45], using 5,5’-dithio-bis(2-nitrobenzoic acid) (DTNB). 10 ml of cells culture were harvested, washed twice with PBS (25 mM potassium phosphate, 150 mM NaCl, pH 7.2) to remove any trace of growth medium, and resuspended in ice-cold 5% perchloric acid. Cells were then broken with glass beads as described above and incubated on ice for 15 min. Cell debris and proteins were pelleted at 18,000 x *g* for 15 min at 4°C and the supernatant neutralized with 100 mM sodium phosphate, 5 mM EDTA, pH 7.5. To assess GSH levels, 600 μM Ellman reagent was added to samples and OD_412_ measured. To assess total glutathione, the neutralized supernatants were incubated at 37°C for 10 min in the presence of 1 U/ml of glutathione reductase and 0.2 mM NADPH and then the GSH content was determined. GSH concentration was determined using a GSH standard curve. Data were expressed as the ratio of GSH to total glutathione content in percentage.

### Antioxidant enzyme activity determination

CAT activity was determined as described by Shangari [46] by measuring the rate of H_2_O_2_ decomposition with the ferrous oxidation. 3 ml of cell culture were harvested and resuspended in hypotonic lysis buffer (10 mM HEPES, 1.5 mM MgCl_2_, 10 mM KCl, 0.5 mM DTT, 0.2 mM PMSF, pH 7.9) and broken as described above. Data were expressed as fold increase with respect to the empty vector strain level.

Superoxide dismutase (SOD) activity was measured using the protocol of enzymatic assay described by Sigma [47]. 3 ml of cell culture were harvested and resuspended in lysis buffer (20 mM phosphate buffer, 5 mM EDTA, 0.2 mM PMSF, pH 7.2) and broken as described above. Data were expressed as fold increase with respect to empty vector strain level.

### Propidium iodide staining

500 μl of cell culture of induction were harvested after 48 h and resuspended in 250 μl of PBS, incubated for 30 min in the dark with 10 μg/ml of propidium iodide (PI). Then, cells were applied to a microscopic slide and observed using a Leica Mod. TCS-SP2 confocal microscope (Leica Microsystem, Wetzlar, Germany). PI fluorescence was excited with the 488 nm line. As a positive control, cells were treated for 15 min with 70% ethanol prior to incubation with PI. Data were expressed as percentage of PI-positive cells.

### Statistical analysis

All experiments were done at least in triplicate. Data are presented as means ± standard error of fold increase or percentage. Values were compared by the Student t test. P < 0.05 was considered significant.

## Results

### A yeast model of ataxin-3 toxicity

**Fig 1:**
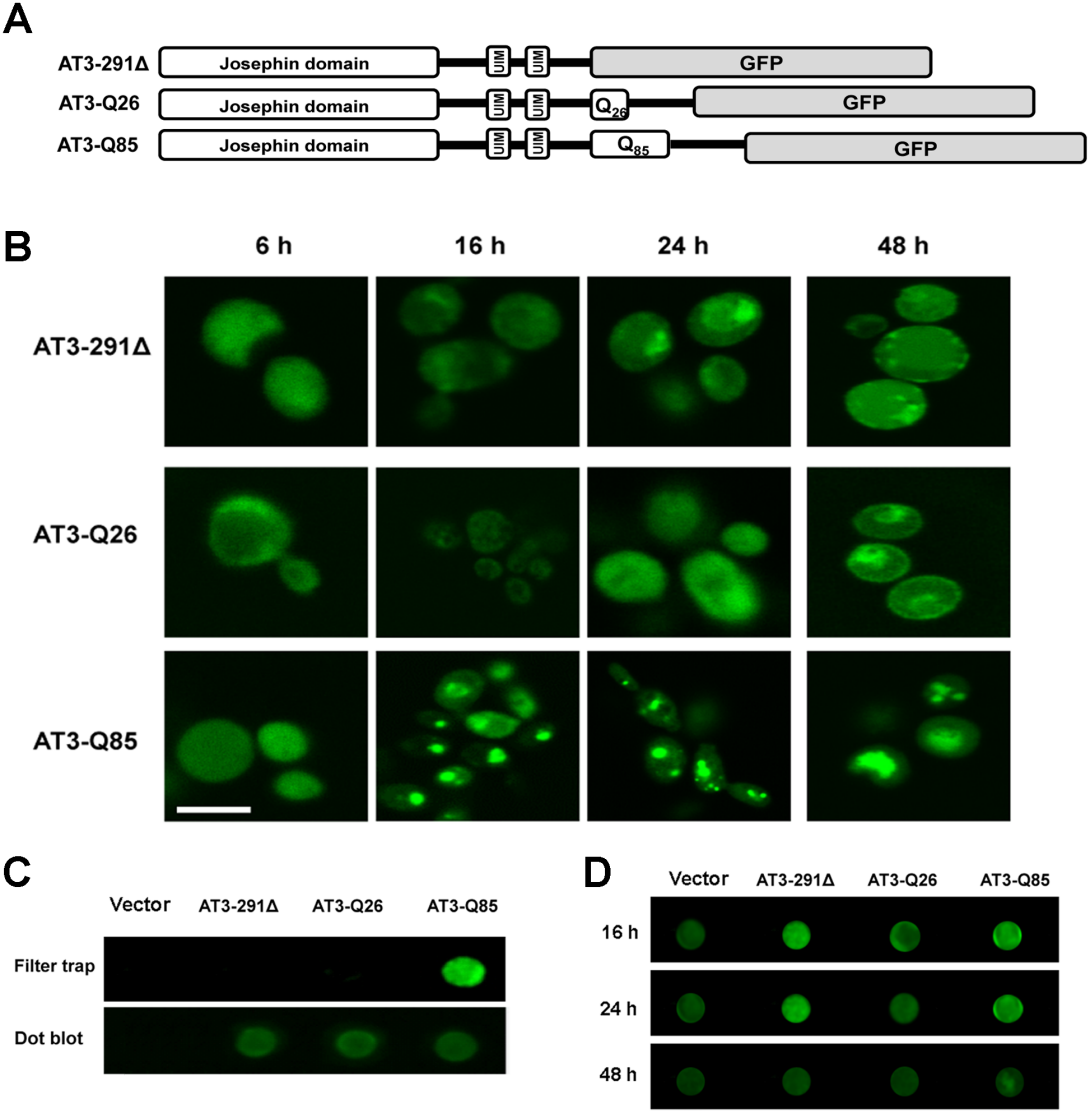
Morphological analysis of AT3 aggregation. A) Sequence and domain organization of the AT3 variants under investigation. UIM: ubiquitin interacting motif, GFP: green fluorescent protein. B) Cells expressing the indicated AT3-GFP fusion proteins were analyzed by fluorescence microscopy (Scale bar: 10 μm) at the indicated times of induction. C) Whole protein extracts of the three strains after 24 h of induction were subjected to filter trap analysis to detect SDS-insoluble aggregates. The immunodecoration was performed using anti-AT3 antibody and anti-rabbit fluorescent secondary antibody. Dot-blot analysis was performed as a loading control. D) Whole protein extracts of *S*. *cerevisiae* strains expressing the AT3 variants were subjected to dot blot at different times after induction and immunodetected using OC antibody and anti-rabbit fluorescent secondary antibody.

To provide insight into the mechanisms of AT3 cytotoxicity in a eukaryotic system, we expressed three variants in *S*. *cerevisiae*, i.e. a wild type and a pathogenic one, carrying 26 (AT3-Q26) and 85 (AT3-Q85) consecutive glutamines respectively, and a variant truncated for a region lying from the beginning of its polyQ stretch to the end of the protein (AT3-291Δ). All constructs were in fusion with GFP at the C-terminus, under the control of the GAL1 promoter and induced by galactose (Fig 1A). Dot blot analysis of whole cell lysates did not show any significant difference in expression among the three variants at different times of induction, nor did the expression level decline significantly with time (Fig. A in S1 File, panel A). SDS-PAGE analysis confirmed the presence of the three variants and their expression levels (Fig. A in S1 File, panel B).

### AT3-Q85 expression leads to the formation of SDS-insoluble aggregates

It has been reported that polyQ expansion in AT3 leads to the formation of intracellular SDS-insoluble aggregates [48]. To check whether this also occurs in our model yeast, we exploited protein constructs in fusion with GFP to monitor their distribution in cells by confocal microscopy analysis (Fig 1B). The results show that the expression of the wild type and of the truncated forms resulted in the appearance at all times from the induction of a largely diffused cytoplasmic fluorescence. In contrast, the expanded variant formed intracellular inclusions starting from 16 h (Fig 1B). Filter trap analysis on whole protein extracts of the three strains at 24 h of induction provided clear evidence in support of a qualitative difference between AT3-Q85 inclusions and those generated by the two other forms, in that the sole AT3-Q85 gave rise to SDS-insoluble aggregates (Fig 1C). To provide more information regarding the nature of such aggregates, we also performed dot blots using OC antibodies that specifically recognize soluble fibrillar oligomers [43]. A strong signal was apparent until 24 h in the case of yeast cell lines expressing AT3-291Δ and AT3-Q85, which faded at the latest time (48 h), suggestive of oligomer evolution into further aggregation forms (Fig 1D). The weak background signal detected under all other conditions, including cells transformed with the vector alone, may be accounted for by amyloidogenic, prion-like yeast proteins, as further outlined in the Discussion.

### AT3-291Δ and AT3-Q85 expression impairs cell growth

**Fig 2:**
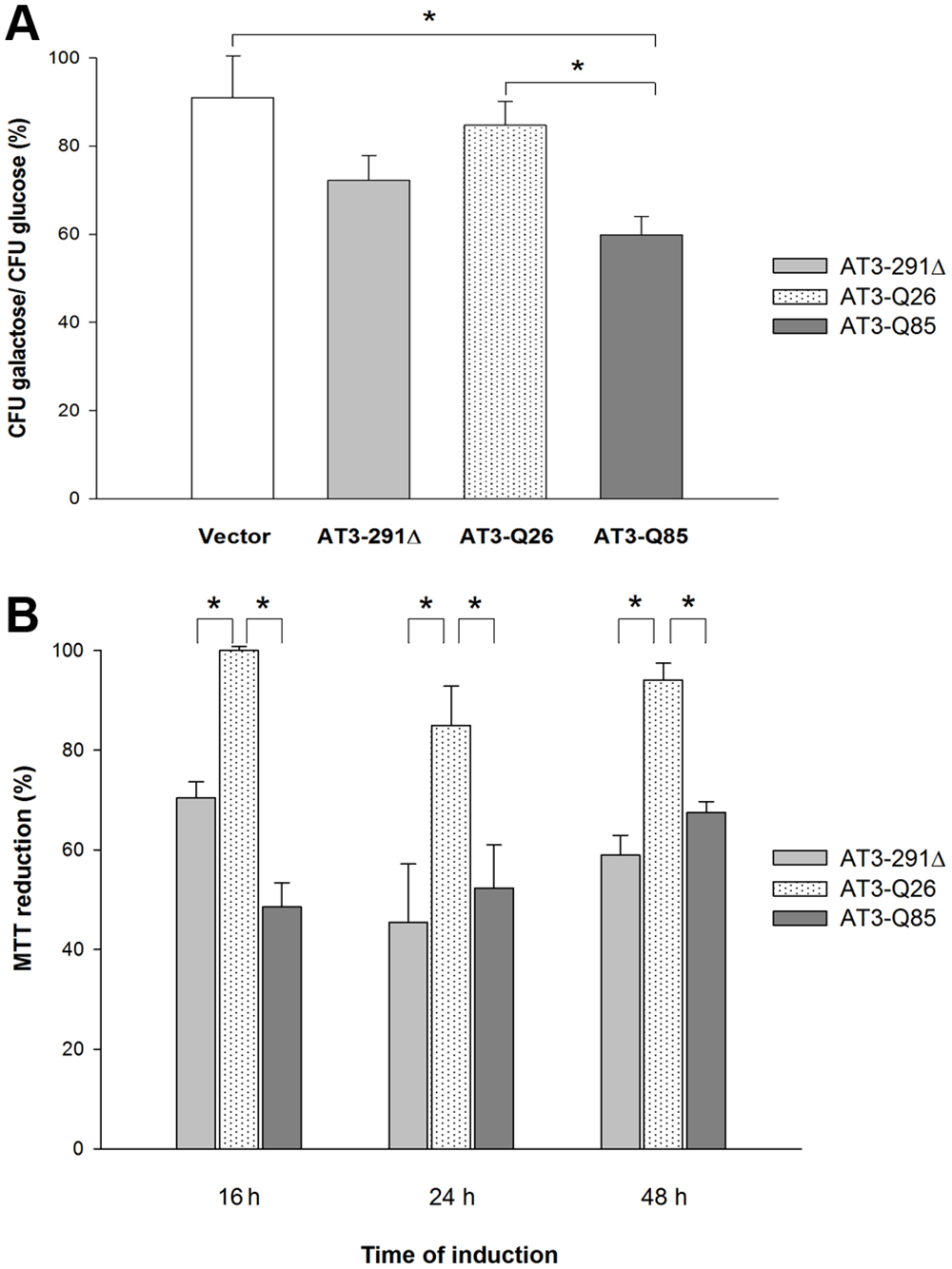
Effect of the AT3 variants expression on cell growth and toxicity. A) Clonogenic assay: about 100 cells from the different cultures were spread onto either glucose or galactose plates and their colony-forming ability expressed as percentage ratio of cells grown under inducing (galactose) *versus* non-inducing (glucose) conditions. Bars represent standard errors and are derived from at least three independent experiments (P < 0.05). B) MTT assay: the experiment was performed on cultures after the indicated induction times. Data are expressed as percentage ratio of MTT reduction *versus* the control (empty vector). Bars represent standard errors and are derived from at least three independent experiments (P < 0.05).

To check whether the expression of the AT3 variants under investigation results in cytotoxicity, we first analyzed their effect on yeast survival rate by a clonogenic assay. Briefly, cells were pre-grown in a medium that repressed expression of the AT3 variants. Then, a fixed amount of cells was plated in parallel onto two different media: without and with inducer (glucose and galactose, respectively) and incubated at 30°C. Their colony-forming ability was determined under either condition (Fig 2A). Results revealed a significant growth-inhibitory effect of AT3-Q85 expression. The AT3-291Δ-expressing strain also showed a decrease in growth capability, although statistically non-significant (Fig 2A).

A significant cytotoxic effect in yeast strains expressing both pathological and truncated variants was also detected by the MTT assay (Fig 2B).

As an alternative approach to assess the effect of the expression of the AT3 variants on yeast survival, we determined the generation time, which yielded results consistent with those of the clonogenic assay. In particular, AT3-Q85 expression resulted in a small but statistically significant increase in duplication time compared to the control (i.e., a yeast cell line transformed with the vector: fig. B in S1 File).

In our previous experimentation, we observed that EGCG and tetracycline reduce AT3 toxicity in both a COS-7 cell line and a transgenic *Caenorhabditis elegans* strain [49]. Thus, as a further validation of our yeast cellular model, we also assayed the effects of these compounds. As expected, they both exerted an almost complete suppression of AT3 toxicity (Fig. C in S1 File).

### AT3-291Δ and AT3-Q85 toxicity is likely to be mediated by the action of the molecular chaperone Hsp104

**Fig 3:**
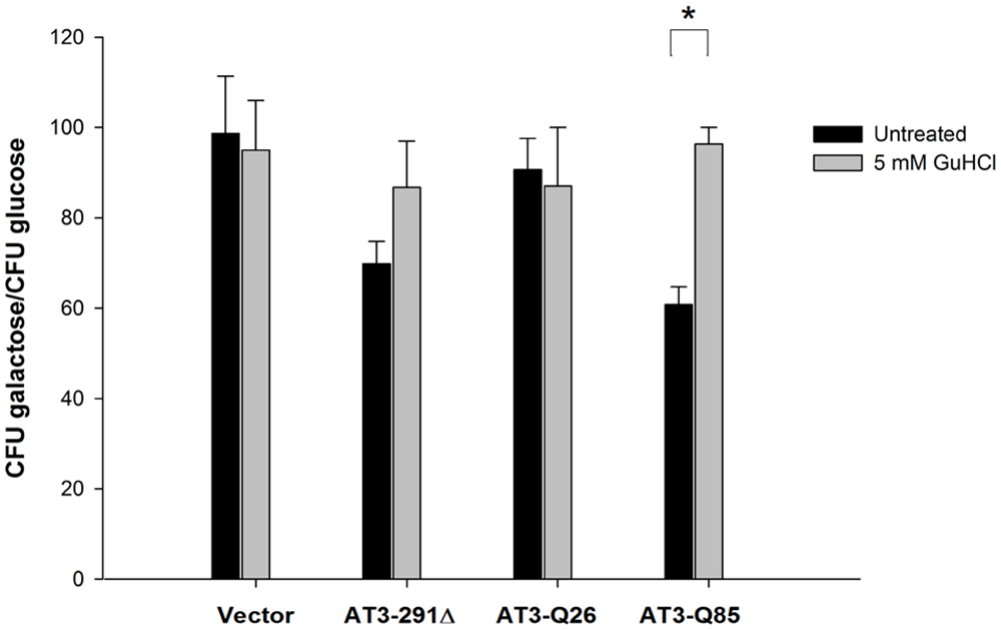
GuHCl restores normal cell growth. About 100 cells grown in the presence or in the absence of 5 mM GuHCl were spread onto either glucose or galactose plates and their colony-forming ability expressed as percentage ratio of cells grown under inducing (galactose) versus non-inducing (glucose) conditions. Bars represent standard errors and are derived from at least three independent experiments (P < 0.05).

A previous report showed that the deletion of the molecular chaperone Hsp104 in yeast significantly suppresses the aggregation of an artificial polyQ-carrying protein (103Q) [32]. Furthermore, it is well known that Hsp104 is also required for prion maintenance [50]. These findings point to the involvement of yeast prion protein in polyQ toxicity, as also supported by its substantial reduction following prion-encoding gene deletion [32,51]. This prompted us to check the effect of GuHCl on AT3 toxicity, as this compound in the millimolar range is capable of inhibiting Hsp104 [32,50,52]. Actually, GuHCl pretreatment almost completely restored wild-type survival rate of both AT3-291Δ- and AT3-Q85-expressing strains (Fig 3), which confirms a role for Hsp104 and possibly for prion protein in AT3 toxicity.

### AT3-291Δ and AT3-Q85 expression induces oxidative stress

**Fig 4:**
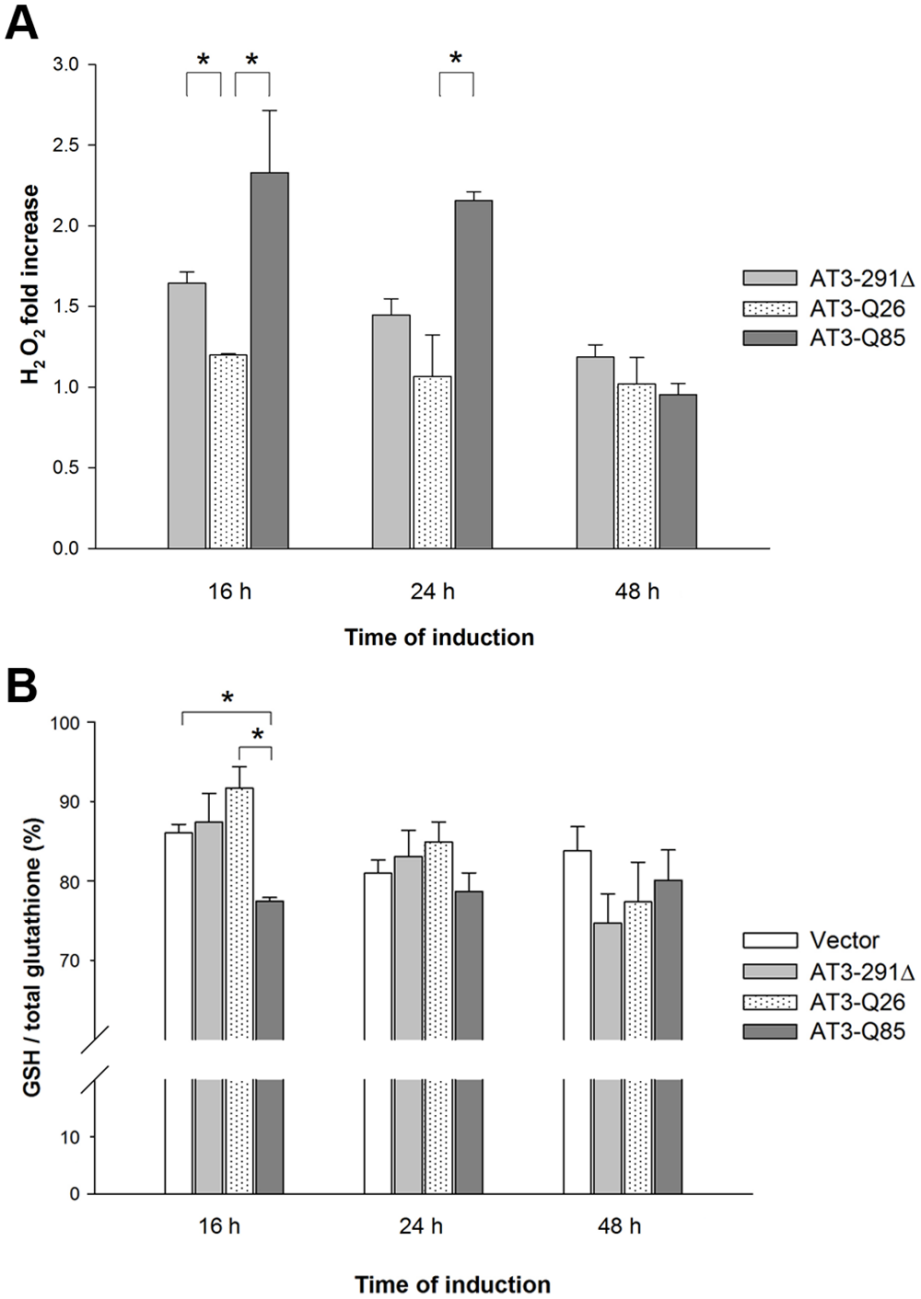
Oxidative stress level in cells expressing AT3 variants. A) ROS levels: intracellular H_2_O_2_ levels were determined using the Red Hydrogen Peroxide Assay Kit. The conversion of red peroxidase substrate to resorufin was determined by measuring the absorbance at 576 nm. Data were expressed as fold increase with respect to the empty vector strain level. Bars represent standard errors and are derived from at least three independent experiments (P < 0.05). B) Glutathione levels: GSH and total glutathione content was determinate using the Ellman reagent. Data were expressed as the ratio of GSH to total glutathione content in percentage. Bars represent standard errors and are derived from at least three independent experiments (P < 0.05).

Besides quantifying cytotoxicity, MTT assay is a marker of mitochondrial stress. To assess whether the growth inhibitory effect observed in the presence of mutant AT3 forms may be ascribed to increased oxidative stress, we first evaluated ROS levels in the three strains. We found that already 16 h after induction, H_2_O_2_ levels were significantly higher in yeast expressing AT3-Q85 and AT3-291Δ compared to AT3-Q26 (1.9 and 1.4 fold increase, respectively) (Fig 4A). At 24 h, the increase was significant only for the strain expressing the expanded form (1.6-fold increase) and, at the latest time, the levels of the three strains were comparable. We then assessed glutathione redox state in the yeast strains at different induction times, by determining the ratio of reduced (GSH) to total glutathione content. Results indicate that at 16 h after induction the ratio in the AT3-Q85 strain underwent a small but statistically significant decrease (by about 1.2 fold), unlike the AT3-291Δ strain that did not show any significant variation at all times assayed (Fig 4B).

### The activity of antioxidant enzymes is increased in strains expressing AT3-291Δ and AT3-Q85

**Fig 5:**
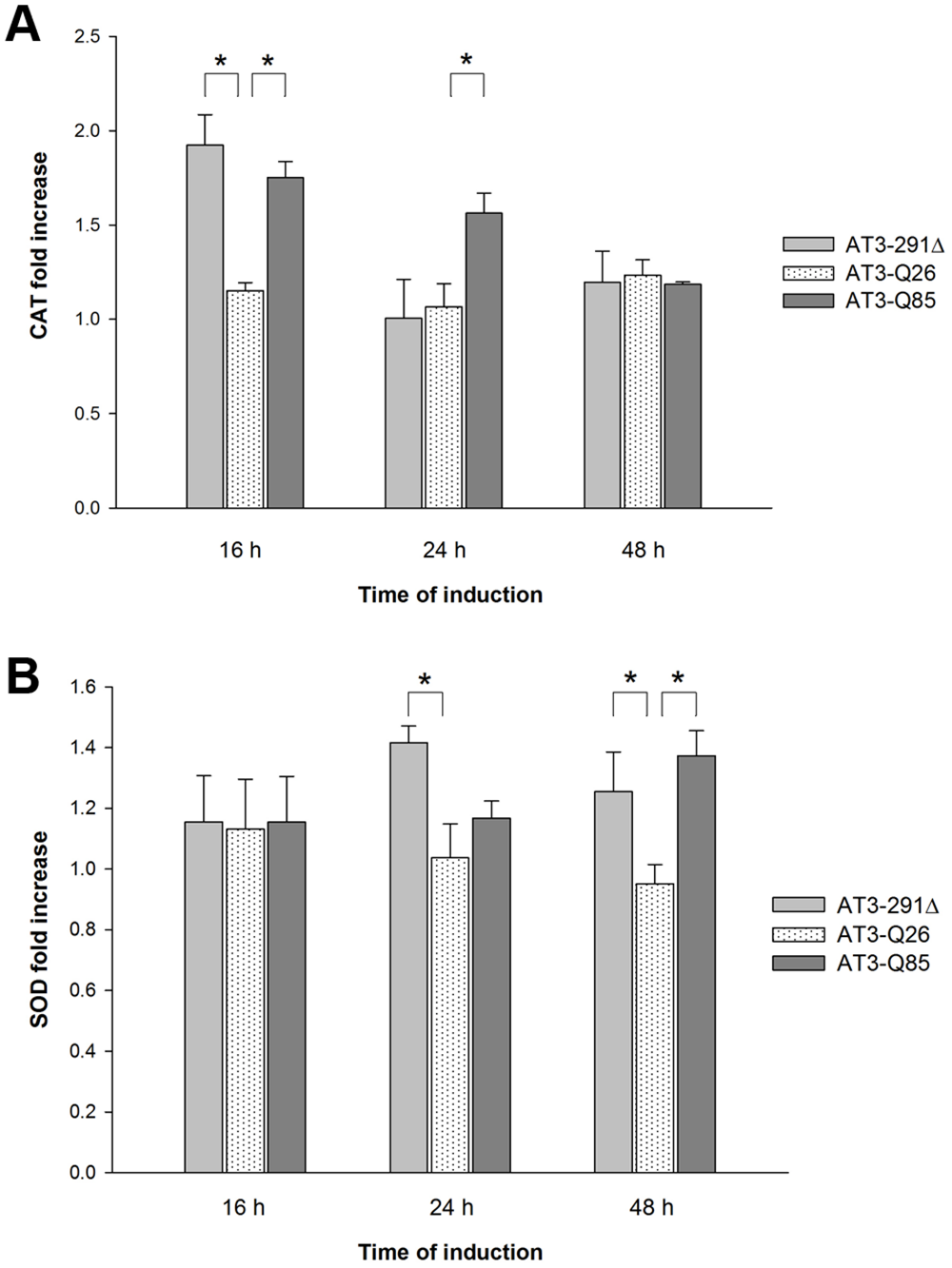
Antioxidant enzyme activity determination. A) CAT activity: the rate of H_2_O_2_ decomposition was determined using the ferrous oxidation assay and absorbance was measured at 560 nm. Data are expressed as fold increase with respect to the empty vector strain level. Bars represent standard errors and are derived from at least three independent experiments (P < 0.05). B) SOD activity was determined as the rate of reduction of oxidized cytochrome *c* at 550 nm. Data are expressed as fold increase with respect to the empty vector strain level. Bars represent standard errors and are derived from at least three independent experiments (P < 0.05).

Enzymatic components in the antioxidant defense system play critical role(s) against oxidative stress. For this reason, we measured CAT and SOD activities to determine whether the detected increase in ROS levels may induce changes in the activity of certain antioxidant enzymes. Our results revealed markedly increased activity of CAT at 16 h of induction in the yeast expressing AT3-Q85 and AT3-291Δ compared to AT3-Q26 (1.5 and 1.7 fold, respectively) (Fig 5A). At 24 h, the increase was significant only for the expanded form (1.5 fold) and at 48 h there were no appreciable differences. As regards SOD, we observed a significant activity increase in the AT3-291Δ strain at 24 and 48 h of induction (1.4 and 1.3 fold, respectively), whereas in the case of AT3-Q85 a significant increase (1.5 fold) was detected only at 48 h of incubation (Fig 5B).

### AT3-Q85 expression affects membrane integrity but does not induce apoptosis

**Fig 6:**
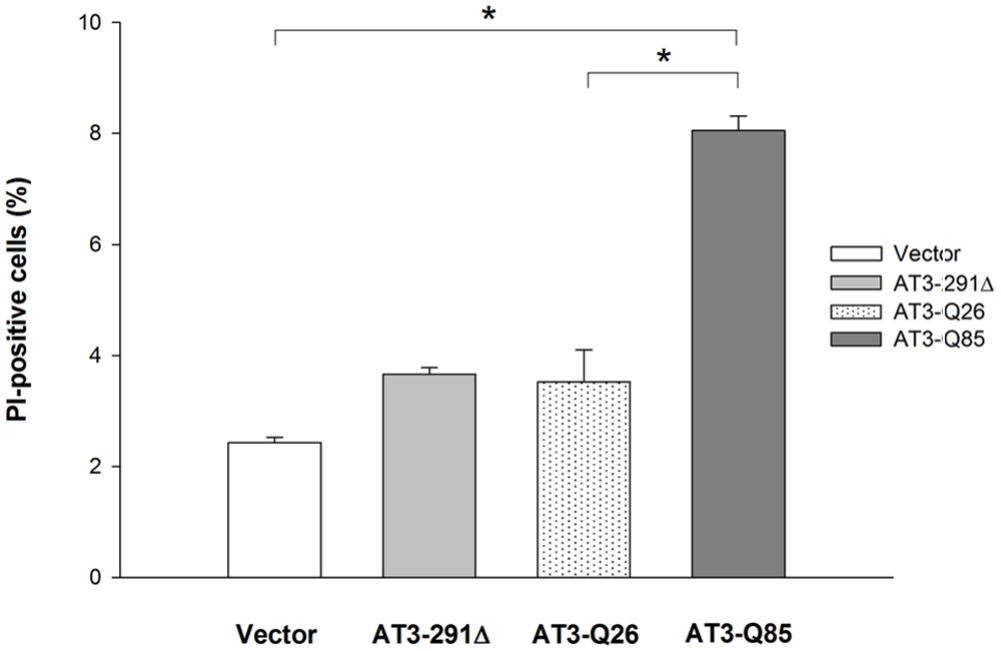
Propidium iodide staining of AT3-expression strains. After a 48-h induction, PI positive cells were counted from > 300 cells from different field views. Bars represent standard errors and are derived from at least three independent experiments (P < 0.05).

To assess whether the expression of the pathological AT3 variant causes membrane damage, we performed propidium iodide (PI) staining, a membrane impermeable dye that binds to double-stranded DNA with resulting fluorescence enhancement. We observed that close to 10% of AT3-Q85-expressing cells took up the dye after 48 h of induction, which is over three-fold compared with the control strain (empty vector) and over two-fold compared with the wild type AT3-expressing strain, indicating loss of plasma membrane integrity and cell necrosis. In contrast, the percentage of PI-positive AT3-Q26- and AT3-291Δ-expressing cells were similar to control cells (Fig 6). Finally, we evaluated cytochrome *c* release from mitochondria in order to verify the presence of apoptotic cells [53]. AT3-expressing strains did not show any significant difference in the amount of cytochrome *c* released at any time (Fig. D in S1 File).

## Discussion

*S*. *cerevisiae* has been long exploited as a model system to investigate the molecular mechanisms underlying neurodegenerative diseases [24]. This became possible thanks to the development of yeast genetic tools, as well as the high conservation of fundamental biological processes and pathways associated with neurodegeneration, including protein folding, cellular trafficking and secretion [54] It is noteworthy that about one-fifth of yeast genes are members of orthologous gene families associated with human diseases [55]. We therefore took advantage of the yeast model to study the mechanisms of toxicity related to the expression of AT3, the protein responsible for SCA3. We employed three AT3 variants: a wild type and an expanded one, carrying 26 (AT3-Q26) and 85 (AT3-Q85) consecutive glutamines, respectively, and a variant truncated for a region lying from the beginning of its polyQ stretch to the end of the protein (AT3-291Δ). All proteins were expressed in fusion with GFP at the C-terminus. By employing such fusion proteins not only could we monitor their aggregation, but also express the expanded AT3 variant, whose authentic form proved to be otherwise refractory to expression. It should be noted, on the other hand, that AT3-Q26 in fusion with GFP did not significantly affect any parameter under investigation, which clearly substantiates the idea that AT3-Q85 toxicity is independent of GFP.

We demonstrated that the expression of the expanded form induces a significant growth-inhibitory effect. Although statistically non-significant, the AT3-291Δ-expressing strain also exerted some effect, supporting the hypothesis that the polyQ-harboring context is also involved in fibrillogenesis and in protein toxicity, as confirmed by the MTT assay. Noteworthy, a toxic effect by AT3 variants truncated in the disordered, C-terminal domain, including the polyQ stretch, was previously demonstrated in both *Escherichia coli* [37] and mice either homozygous or heterozygous for the truncated AT3-259Δ, which developed severe motor coordination dysfunction and altered behavior, followed by premature death [56]. Thus, the capability of truncated variants to trigger toxic effects is a well-established phenomenon that develops irrespective of the biological milieu.

A hallmark of SCA3 pathology is the presence of amyloid aggregates in the brain. Through fluorescence microscopy analysis, we showed the formation of aggregation *foci* in the AT3-Q85-expressing strain starting from 16 h after induction. This phenotype should be accounted for by the intrinsic properties of the protein, rather than by its overexpression, as substantiated by the fact that AT3-Q26- and AT3-291Δ-expressing strains did not show any such aggregates, although the three variants were expressed at similar levels. Filter trap analysis showed that SDS-insoluble aggregates only arose from the expanded variant after a 24 h-induction (Fig 1C), whereas at 16 h no such aggregates were generated by any of the AT3 forms (data not shown), in agreement with a previous study [37]. This suggests that intracellular aggregates observed at 16 h are pre-fibrillar species rather than SDS-insoluble mature fibrils. Thus, the fact that the aggregation patterns of AT3-291Δ and AT3-Q85 are somewhat different from each other, suggests that the toxicity mechanisms must also differ, at least to some extent.

To further clarify the mechanisms of AT3 toxicity, we checked the effect of GuHCl on cell growth using the clonogenic assay. GuHCl is a well-known inhibitor of the yeast chaperone Hsp104 [32,50,52], which in turn has been implicated in prion maintenance [50]. GuHCl treatment almost completely restored normal cell growth in AT3-Q85 and AT3-291Δ strains. This strongly supports the idea that Hsp104 promotes AT3 aggregation and toxicity by propagating prion forms of yeast proteins, such as [PSI^+^] or [PIN^+^] [32,50,52]. We suggest that such forms might act to seed AT3 aggregation, thus substantially accelerating the process and boosting toxicity. Noteworthy, the toxicity of the truncated form, although devoid of the polyQ tract, was also mitigated.

MTT assays highlighted a significant toxic effect induced by the expanded and truncated variants, unlike the wild type. Thus, seeking for mechanisms of toxicity, we assessed possible oxidative stress. Indeed, it is known that amyloid aggregates may boost reactive oxygen species (ROS), which in turn results from mitochondrial dysfunction [57]. We found that, already 16 h after induction, H_2_O_2_ levels were significantly higher in yeast expressing AT3-Q85 and AT3-291Δ, compared to AT3-Q26. At 24 h, the increase was significant only for the expanded form and, at the latest time, the levels of the three strains were comparable. However, this pattern may due to culture aging, which implies progressive ROS-induced cellular damage in all organisms [58], including yeast [59]. Actually, H_2_O_2_ level underwent a substantial increase at the latest time (48 h) independent of the toxic effects of the AT3 variants, as detected in the empty vector strain (data not shown). This likely overshadowed the effects related to protein toxicity, which prevented the possibility to investigate them at the latest time. Thus, our results provide evidence that oxidative stress is implicated in AT3 toxicity and suggest its involvement in the early events occurring at the onset of disease. Alterations in the antioxidant defense system were also demonstrated by the imbalance of the ratio reduced (GSH) to total glutathione. In fact, this was significantly lower in cells expressing AT3-Q85 with respect to the two other strains, whilst total glutathione was not affected by protein expression (data not shown).

To further dissect the mechanisms that mediate the altered redox status in our model, we examined the cellular enzymatic defense system against oxidative stress by assaying CAT and SOD levels. Our results revealed markedly increased activity of CAT at 16 h after induction in the yeast expressing AT3-Q85 and AT3-291Δ compared to AT3-Q26. At 24 h, the increase was significant only for the expanded form. Similar to the case of H_2_O_2_, SOD levels at 48 h also substantially increased in the control strain (empty vector), and concurrently no apparent toxic effect of protein expression was detectable at that time. In contrast, SOD levels kept essentially constant in the control strain, but significantly increased in the AT3-291Δ strain at both 24 and 48 h of induction, whereas in the case of AT3-Q85 the increase was significant at 48 h only. Previous investigations evaluated oxidative stress biomarkers in SCA3 mammalian cells [60] and patients [61]. In either case, a drop in reduced thiols was detected, in keeping with our results. However, data regarding the level of antioxidant enzymes in diseased cells/patients compared to the controls are conflicting, as CAT was increased in patients, similar to our yeast model, whereas in mammalian cells both CAT and SOD dropped compared to the controls. We have no obvious explanation for such discrepancy, although the decline in CAT and SOD activity in cell cultures might be accounted for as a consequence of oxidative damage [60].

Summarizing, in the AT3-Q85-expressing strain an increase in ROS levels was paralleled by a fast GSH drop and a significant increase in CAT activity, whereas SOD activity increased only after 48 h of induction. The observed pattern may be possibly accounted for by the failure of AT3-Q85 and AT3-291Δ strains to effectively degrade excess of H_2_O_2_ by thiol groups, although there seems to be a compensatory mechanism that increases CAT and SOD levels compared to the control. Moreover, these data suggest that the appearance of SDS-soluble aggregates at 16 h (Fig 1B and 1C) induces mitochondrial damage, increases in ROS species and a consequent imbalance of the antioxidant defense system. These findings are in line with the hypothesis that oligomeric and pre-fibrillar species formed at the initial stages of the aggregation process are those responsible for cellular toxicity. Moreover, expression of AT3-291Δ also showed a toxic effect, albeit milder than that of the expanded form, which also is likely to be related to its proven capability to form oligomeric species [37].

Another toxicity mechanism involved in neurodegenerative diseases is the direct interaction of amyloid aggregates with lipid membranes and their consequent permeabilization [62–64]. This prompted us to perform PI-staining analyses. Our data show that close to 10% of AT3-Q85-expressing strain underwent loss of plasma membrane integrity and cell necrosis after 48 h of induction. In contrast, neither the AT3-291Δ strain nor the one expressing wild-type protein showed any necrosis marker. Based on our data, the causal relationship between the toxic effects resulting from AT3-Q85 expression and membrane damage cannot be precisely established. It is possible that both ROS and AT3 oligomeric forms affect membrane integrity, as observed in previous reports [65–67].

No apotosis was observed in our yeast model using the cytochrome c assay, although we previously reported the capability of the aggregates formed *in vitro* by AT3-291Δ and AT3-Q55 to induce apoptosis in rat cerebellar granule neurons when added to cell medium. This apparent contradiction is probably due to different experimental strategies, as in the yeast model the protein was expressed in the intracellular environment and targeted by the cellular defense system, whereas in the case of rat neurons it was added to the medium.

In conclusion, this work shows that *S*. *cerevisiae* is a suitable model to study the mechanisms of SCA3 pathogenesis. Actually, not only did our investigations in yeast detect the toxicity of both expanded and truncated AT3, in keeping with data collected in other cellular milieus [37,65] and animal models [56], but could also reveal differences in behavior between the two forms, in terms of aggregation patterns *in vivo* and toxicity. Thus, it is plausible that such differences also rely upon qualitatively different cellular mechanisms, which might justify the milder phenotype of the truncated form compared to the expanded one. In any case, our results strongly support the idea that AT3 toxicity could be correlated with the formation of oligomeric and pre-fibrillar aggregates that cause oxidative stress in the short-term, whereas long-term effects might affect cell membrane integrity, at least in the case of the expanded form.

## Supporting Information

S1 File This file contains methods, figures and captions of: A) expression level quantification of the AT3 variants; b) growth rates assessed by duplication time; c) the effects of EGCG and tetracycline on colony-forming abilities of AT3 expressing strains; d) cytochrome C release assay.
(ZIP)
