# Supplementary material for: The Toxic Effects of Pathogenic Ataxin-3 Variants in a Yeast Cellular Model
Source: PLoS One. 2015 Jun 8;10(6):e0129727. doi: 10.1371/journal.pone.0129727 (PMC4460139; doi:10.1371/journal.pone.0129727)
Supplement: S1 File — This file contains methods, figures and captions of: A) expression level quantification of the AT3 variants; b) growth rates assessed by duplication time; c) the effects of EGCG and tetracycline on colony-forming abilities of AT3 expressing strains; d) cytochrome C release assay. (ZIP) [file pone.0129727.s001.zip › S1_Supporting information.docx]

**S1 File: Supporting information**

**Supplemental Materials and methods**

*SDS-PAGE and western blot analyses*

Whole protein extracts, obtained by glass beads rupture as described in Material and methods, were analyzed in 12% SDS–polyacrylamide gel and AT3 was revealed by western blotting using an anti-AT3 Z46 rabbit polyclonal antibody [42]. Membranes were incubated for 60 min at room temperature with the antibody at 1:5000 dilution in blocking solution. After incubation in primary antibody, membranes were rinsed thrice in 0.1% Tween in PBS, each time for 10 min, and incubated for 60 min in anti-rabbit fluorescent secondary antibody (Donkey anti-rabbit IRDye 800 CW, Li-Cor, Lincoln, USA) at 1:15000 dilution. Membranes were imaged using a LiCor Odyssey Fc scanner.

*Duplication time*

Cells were grown in selective medium containing 2% glucose, washed thrice in sterile water and diluted to an OD_600_ of 0.1 in selective medium containing 2% galactose as an inducer. OD_600_ was monitored at 30-min intervals (starting from 16 h after induction) after a ten-fold dilution in water.

*Cytochrome c release quantification*

Cytosolic fractions were isolated essentially as described in [53] with minor modifications. 100 ml of cell culture were incubated with 1 mg/g of cell weight of lyticase (Sigma-Aldrich, St. Louis, MO, USA) for 1 h at 30 °C in SIB buffer (1.2 M sorbitol, 20 mM potassium phosphate, pH 7.4) to obtain spheroplasts*.* Spheroplasts were harvested by centrifugation for 5 min at 3.000 x *g,* washed once with SIB buffer and resuspended in 1 ml of ice-cold MIB buffer (0.6 M mannitol, 20 mM HEPES– KOH, pH 7.4)*.* Spheroplasts were homogenized on ice by 15 strokes in a tight-fitting Dounce homogenizer*.* Homogenates were centrifuged for 20 min at 12,000 x *g.* The supernatant was further centrifuged for 10 min at 12,000 x *g* to reduce mitochondrial contamination. 40 μg of cytosolic extract was loaded onto 16% SDS–polyacrylamide gel and cytochrome *c* revealed by western blotting using a polyclonal anti-cytochrome *c* antibody (H-104, Santa Cruz Biotechnology, Santa Cruz, CA, USA), then probed with anti-rabbit fluorescent secondary antibody (Donkey anti-rabbit IRDye 800 CW, Li-Cor, Lincoln, USA). As a mitochondrial contamination control, we also revealed the mitochondrial protein acetohydroxyacid reductoisomerase (Ilv5p) using a polyclonal antibody kindly provided by Dott. Sergio Giannattasio (Institute of biomembrane and bioenergetics, CNR Bari, Italy) [53], then probed with anti-mouse fluorescent secondary antibody (Donkey anti-mouse IRDye 680 CW, Li-Cor, Lincoln, USA). Nitrocellulose membranes were imaged using a LiCor Odyssey Fc scanner and densitometric analyses were performed using Image Studio software (Li-Cor, Lincoln, USA). Data were expressed as fold increase with respect to the empty vector strain cytochrome *c* level after normalization based on the amount of Ilv5p in each sample.

**Supplemental information Legends**

**Fig. A. AT3 variants and their expression levels.** A) Dot blot analysis of AT3. Whole protein extracts of *S. cerevisiae* strains expressing the AT3 variants were subjected to dot blot at different times after induction and immunodetected using anti-AT3 antibody and anti-rabbit fluorescent secondary antibody. B) Western blot analysis of AT3 expression levels. Whole protein extracts of *S. cerevisiae* strains expressing the AT3 variants at 24 h after induction were subjected to SDS-PAGE and western blotted using anti-AT3 antibody and anti-rabbit fluorescent secondary antibody.

**Fig. B. Effect of the AT3 variants expression on cell growth, as assessed by generation times.** OD_600_ of the different *S. cerevisiae* strains was monitored at 30-min intervals during the exponential phase. Bars represent standard errors and are derived from at least three independent experiments (P < 0.05).

**Fig. C. EGCG and tetracycline mitigate AT3 toxicity.** About 100 cells grown in the presence or in the absence of either 100 µM tetracycline or 100 µM EGCG were spread onto either glucose or galactose plates and their colony-forming ability expressed as percentage ratio of cells grown under inducing (galactose) versus non-inducing (glucose) conditions. Bars represent standard errors and are derived from at least three independent experiments (P < 0.05).

**Fig. D. Western blot analysis of cytochrome *c* release.** Cytosolic fractions of *S. cerevisiae* strains expressing the AT3 variants at different times after induction were subjected to SDS-PAGE and western blotted using anti-cytochrome *c* or anti-Ilv5p antibody. Data are expressed as fold increase with respect to the empty vector strain cytochrome *c* level after normalization based on the amount of Ilv5p in each sample. Bars represent standard errors and are derived from at least three independent experiments.
