# Supplementary figures and images for: The Toxic Effects of Pathogenic Ataxin-3 Variants in a Yeast Cellular Model
Source: PLoS One. 2015 Jun 8;10(6):e0129727. doi: 10.1371/journal.pone.0129727 (PMC4460139; doi:10.1371/journal.pone.0129727)

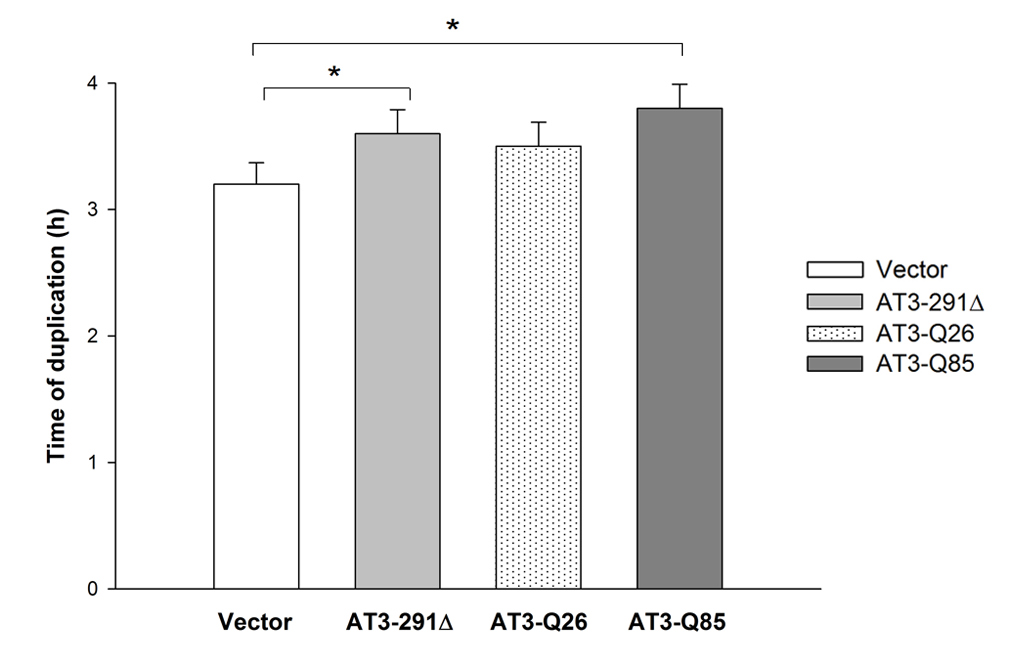

Supplement: S1 File — This file contains methods, figures and captions of: A) expression level quantification of the AT3 variants; b) growth rates assessed by duplication time; c) the effects of EGCG and tetracycline on colony-forming abilities of AT3 expressing strains; d) cytochrome C release assay. (ZIP) [file pone.0129727.s001.zip › Fig B.tif]

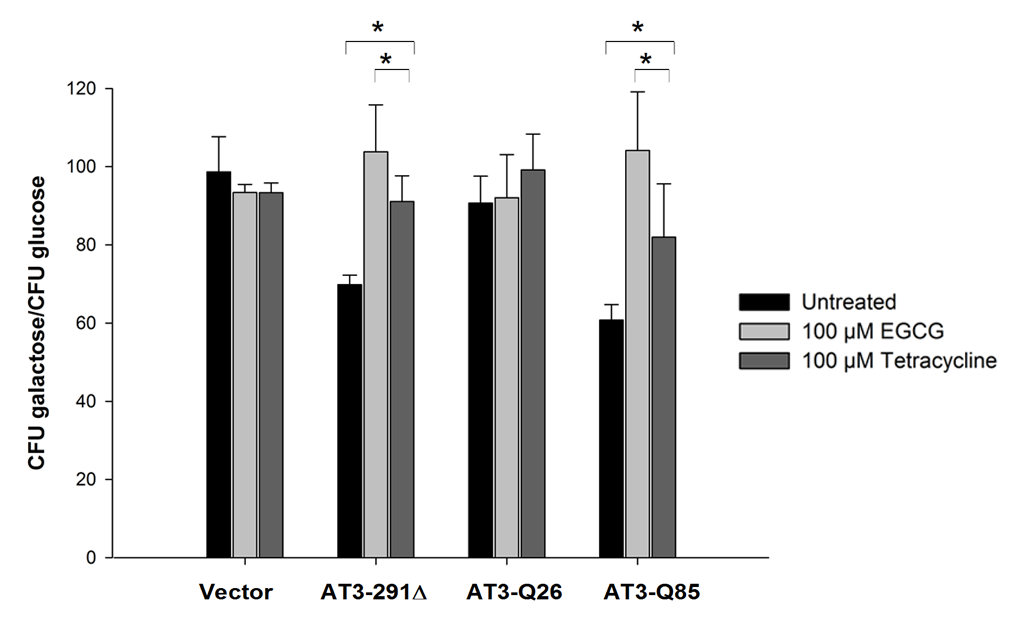

Supplement: S1 File — This file contains methods, figures and captions of: A) expression level quantification of the AT3 variants; b) growth rates assessed by duplication time; c) the effects of EGCG and tetracycline on colony-forming abilities of AT3 expressing strains; d) cytochrome C release assay. (ZIP) [file pone.0129727.s001.zip › Fig C.tif]

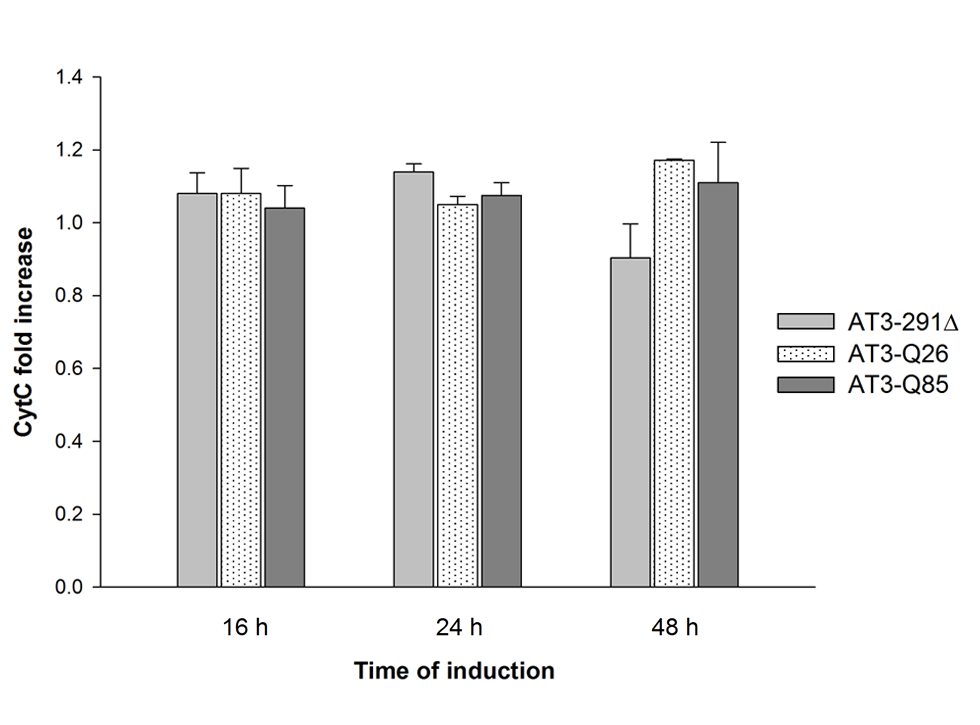

Supplement: S1 File — This file contains methods, figures and captions of: A) expression level quantification of the AT3 variants; b) growth rates assessed by duplication time; c) the effects of EGCG and tetracycline on colony-forming abilities of AT3 expressing strains; d) cytochrome C release assay. (ZIP) [file pone.0129727.s001.zip › Fig D.tif]

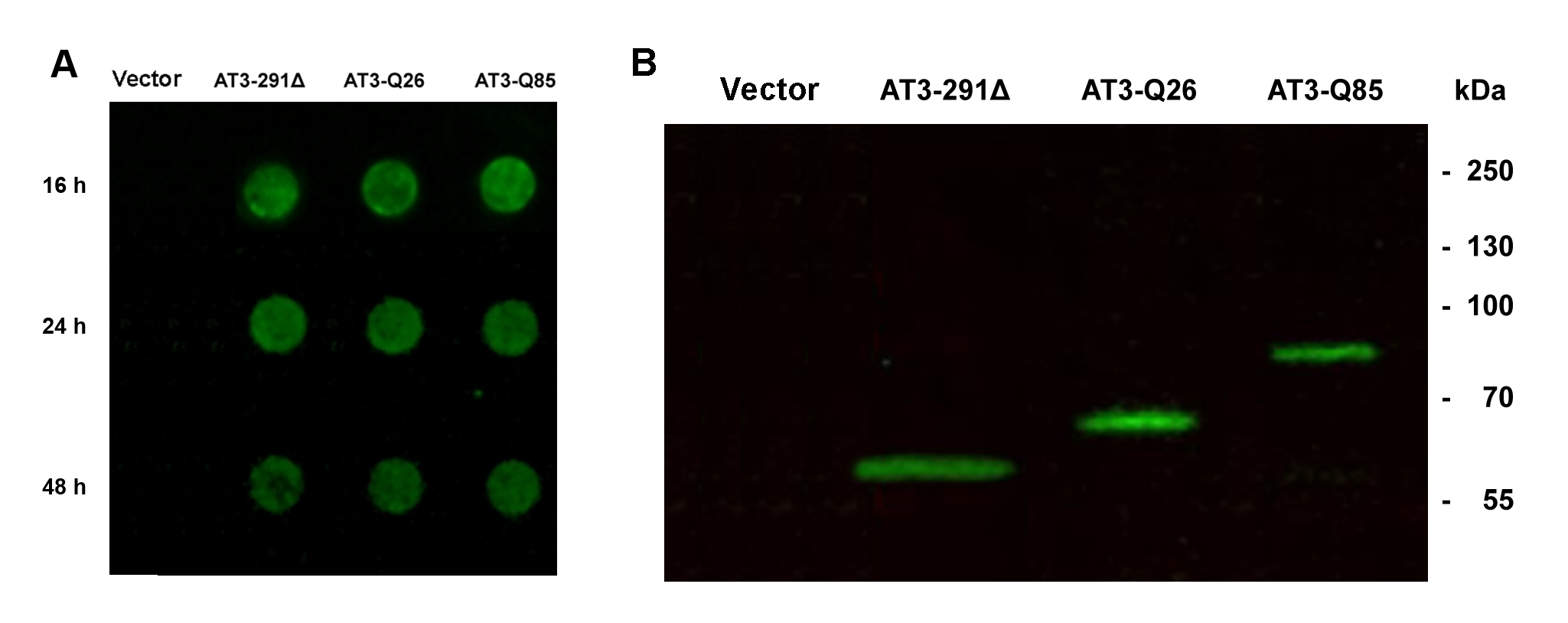

Supplement: S1 File — This file contains methods, figures and captions of: A) expression level quantification of the AT3 variants; b) growth rates assessed by duplication time; c) the effects of EGCG and tetracycline on colony-forming abilities of AT3 expressing strains; d) cytochrome C release assay. (ZIP) [file pone.0129727.s001.zip › Fig A.tif]
